# Exercise Habits During Ramadan Among Healthcare Professionals: A Cross-Sectional Observational Study From Jeddah, Saudi Arabia

**DOI:** 10.7759/cureus.49665

**Published:** 2023-11-29

**Authors:** Hala Mosli

**Affiliations:** 1 Endocrinology, King Abdulaziz University, Jeddah, SAU

**Keywords:** healthcare professionals, ramadan, exercise, public health, physical activity

## Abstract

Background

Fasting during Ramadan may affect several habits, including physical activity levels. Therefore, the present study was designed to assess changes in exercise habits among healthcare providers (HCPs) during Ramadan in Jeddah, Saudi Arabia. This study aimed to evaluate the factors associated with changes in exercise habits during Ramadan.

Methodology

This cross-sectional, observational study included HCPs working in Jeddah, Saudi Arabia. Data were collected using an electronic self-administered questionnaire between March and April 2020. McNemar’s test was used to assess the difference between exercise habits during Ramadan and the rest of the year. Pearson’s chi-square test was used to explore the factors affecting the rate and intensity of exercise during Ramadan. P-values less than 0.05 were considered statistically significant.

Results

A total of 89 HCPs were enrolled in the study. Of these, 64% (n = 57) were female, and 67.4% (n = 60) worked in governmental hospitals. Of these, 58.4% (n = 52) had moderate physical activity, and 41.6% (n = 37) had low exercise intensity during Ramadan. The percentage of low-intensity exercises increased to 52.8% (n = 47). Almost one-third of the HCPs who usually performed moderate or severe-intensity exercise decreased their intensity significantly to a low level. In addition, 10.8% (n = 4) of respondents upgraded their exercise intensity from low to moderate or severe levels during Ramadan. Meanwhile, Ramadan had no significant impact on the usual exercise rate.

Conclusions

The present study demonstrated the reduction in the intensity of exercise among HCPs during Ramadan without changing the usual exercise rate.

## Introduction

Physical activity is considered an essential habit for better health [1]. Routine physical activity is associated with a significant reduction in premature mortality risk and can help prevent over 25 chronic medical conditions [2,3]. In Saudi Arabia, the World Health Organization reported that physical inactivity is common among adults, youth, and children [4]. It is essential to ensure a good level of physical activity among healthcare providers (HCPs), as it significantly reflects on patient counseling and the quality of provided care [5]. Exercise habits and physical activity are affected by different factors, such as behavioral, social, and environmental factors [6]. Ramadan fasting may lead to different changes in lifestyle and habits, such as the hours of work and exercise habits [7,8]. During Ramadan, Muslims change their lifestyle and physical activities. Religious fasting leads to spiritual growth and can also improve physical health and aid in weight loss [9-11]. However, results from a previous survey conducted among Saudi families showed that a significant proportion of the population (two-thirds) reported weight gain during Ramadan, and one-third reported decreased physical activity levels, which could significantly impact their daytime productivity [12]. Recognizing the impact of Ramadan habits on physical activity can help in improving physical activity levels among HCPs [13]. Thus, this study aimed to assess the level of physical activity during Ramadan among an important sector of the population, healthcare practitioners.

## Materials and methods

Study design

This cross-sectional study was conducted between March and April 2020 in Jeddah, Saudi Arabia using an electronic self-administered questionnaire to assess the level of physical activity among HCPs during Ramadan. Before data collection (during the COVID-19 period), a verbal exemption was obtained from the institutional review board (IRB). The study received an ethical exemption letter from the National Committee of Bioethics at King Abdulaziz City for Science and Technology (IRB registration number: HA-02-J-008, reference number for exemption: 31-23 on 08-05-2023).

Study population

The study was conducted among HCPs at two time frames in Jeddah. The participants received information about the study before choosing to fill in the questionnaire and they agreed to it.

Data collection

The questionnaire consisted of three sections. The first section gathered information on participants’ demographic characteristics, including age, gender, and marital status. The second section included questions pertaining to participants’ regular exercise habits throughout the year. This included the number of exercise hours per week, the intensity of exercise (as assessed by the responder), and the type of preferred exercise. The third section addressed exercise habits, specifically during the month of Ramadan. This included the number of exercise hours per week, the intensity of exercise, the type of preferred exercise, the change in body weight, exercise time (before/after Iftar), and barriers to exercise. The questionnaire was validated by two experts in the field. Before data collection, a pilot study on 30 subjects was conducted to ensure the clarity of the questions.

Statistical analysis

The data were entered into a Microsoft Excel version 2020 sheet (Microsoft Corp., Redmond, WA, USA) and cleaned. SPSS version 26 (IBM Corp., Armonk, NY, USA) was used for data analysis. Frequencies and percentages were used for descriptive statistics for categorical data. McNemar’s test was used to assess the difference between the exercise habits among participants during Ramadan and the rest of the year. Pearson’s chi-square test was used to find the association between independent variables and the rate and intensity of exercise during Ramadan. P-values less than 0.05 were considered statistically significant. The level of type I error was set at 0.5 and that of type II error was set at 0.1. The sample size was calculated considering a 10% margin error, 95% confidence level, and an expected response distribution of 33% (the proportion of subjects with a change in their physical activity level during Ramadan) [12], resulting in a minimum required sample size of 85 HCPs.

## Results

A total of 89 HCPs were included in the study. Two-thirds of the participants were females (64%, n = 57) and worked in governmental hospitals (67.4%, n = 60). About 50.6% (n = 45) of the participants were aged 31 to 40 years old. Additionally, 40.4% (n = 36) were physicians. Most respondents were married (77.5%, n = 69) and had no chronic medical illness (79.8%, n = 71). Moreover, more than half of the individuals had moderate physical activity (58.4%, n = 52) and performed a body composition analysis (52.8%, n = 47). All data are illustrated in Table 1.

**Table 1:** Demographic characteristics of participants. The data are represented as Numbers (n) and percentages. *: Other specialties included pharmacists, dentists, and psychiatrists.

Parameters	Category	Count (n = 89)	Percentage
Age	20–30 years	9	10.1
	31–40 years	45	50.6
	41–50 years	23	25.8
	51–60 years	7	7.9
	More than 60 years	5	5.6
Gender	Male	32	36
	Female	57	64
Marital status	Single	15	16.9
	Married	69	77.5
	Divorced	3	3.4
	Separated	2	2.2
Specialty	Physician	36	40.4
	Surgeon	12	13.5
	Nurse	8	9
	Physical therapist	8	9
	Others*	25	28.1
Workplace	Governmental hospital	60	67.4
	Private practice	13	14.6
	Both	13	14.6
	Retired	3	3.4
Chronic medical illness requiring pharmacological therapy	Yes	18	20.2
	No	71	79.8
Usual exercise activity	Sedentary	16	18
	Moderately active	52	58.4
	Very active	18	20.2
	Athletic	3	3.4
Having body composition analysis using a commercially available device	Yes	47	52.8
	No	38	42.7
	Don’t know	3	3.4

This study demonstrated the usual exercise habits of participants throughout the year (Table 2). The study showed that about 61.8% (n = 55) of individuals performed physical exercise for three hours or less per week. Additionally, 41.6% (n = 36) of respondents performed low-intensity exercise on usual days. The participants’ most preferred types of exercise were reported to be strength and endurance (33.7%, n = 30), followed by cardiorespiratory (32.6%, n = 29).

**Table 2:** Usual exercise habits during Ramadan and the rest of the year (ROY). The data are represented as numbers (n) and percentages.

Parameters	Category	Count (n = 89)	Percentage
Rest of the year			
Exercise duration per week	Less than 1 hour	29	32.6
	1–3 hours	26	29.2
	3–5 hours	17	19.1
	5–7 hours	9	10.1
	More than 7 hours	8	9
The intensity of exercise	Low	37	41.6
	Moderate	22	24.7
	Vigorous	7	7.9
	Mixture of intensity	23	25.8
Type of exercises preferred	Cardiorespiratory	29	32.6
	Strength and endurance	30	33.7
	Flexibility and conditioning	16	18
	Yoga	1	1.1
	Walking	6	6.7
	Squash	2	2.2
	Tennis	3	3.4
	All of the above	2	2.2
During Ramadan			
Exercise duration per week	Less than 1 hour	32	36
	1–3 hours	32	36
	3–5 hours	12	13.5
	5–7 hours	6	6.7
	More than 7 hours	7	7.9
The intensity of exercise	Low	47	52.8
	Moderate	25	28.1
	Vigorous	3	3.4
	Mixture of intensity	14	15.7
Type of exercises preferred	Cardiorespiratory	33	37.1
	Strength and endurance	24	27
	Flexibility and conditioning	23	25.8
	Yoga	1	1.1
	Walking	4	4.5
	Tennis	3	3.4
	All the above	1	1.1
Exercise time	Before Iftar	36	40.4
	After Iftar	31	34.8
	Alternating	22	24.7
Change in weight	Gain weight	16	18
	Lose weight	27	30.3
	No change	46	51.7
Exercising barriers	Lack of energy and motivation	2	2.2
	Lack of interest	18	20.2
	Limited availability of exercise facilities during your preferred time	13	14.6
	Prefer to focus on other priorities	15	16.9
	Lockdown due to COVID-19	3	3.4
	Time restriction	35	39.3
	None	3	3.4

Regarding exercise habits during Ramadan, about 72% (n = 64) of respondents exercised for three hours or less per week, and 52.8% (47) performed a low-intensity exercise. Furthermore, cardiorespiratory (37.1%, n = 33), followed by strength and endurance (27%, n = 24), were the most preferred exercises during Ramadan. Less than half of the participants (40.4%, n = 36) exercised before Iftar. Additionally, about 39.3% (n = 35) of individuals thought that time restriction was the main reason for the change in exercise during Ramadan. Moreover, 51.7% (n = 46) of individuals confirmed that they did not gain weight during Ramadan. All data are demonstrated in Table 2.

This study illustrated no significant difference between the duration of physical exercise during Ramadan and the rest of the year (p = 0.05). For instance, the largest proportion of the study population (n = 25, 28.1%) exercised for less than one hour/week during Ramadan and the rest of the year. Additionally, 24 (27.0%) individuals usually exercised more than three hours per week during Ramadan and the rest of the year. Data are illustrated in Table 3.

**Table 3:** Comparison of physical exercise duration during Ramadan and the rest of the year (ROY). The data are represented as numbers (n) and percentages. The association is presented as p-values.

Ramadan	ROY			P-value
Parameters	Less than 1 hour	1-3 hours	More than 3 hours	0.05
Less than 1 hour	25 (28.1%)	6 (6.7%)	1 (1.2%)	
1–3 hours	4 (4.5%)	19 (21.3%)	9 (10.1%)	
More than 3 hours	0 (0.0%)	1 (1.2%)	24 (27.0%)	

This study illustrated the significant statistical association between exercise intensity during Ramadan and the rest of the year with a p-value of 0.009. Almost one-third (34.5%, n = 10) of participants who usually performed moderate or vigorous exercise decreased their exercise intensity to a low-intensity level. Only 10.8% (n = 4) of respondents upgraded their exercise intensity from low to moderate or vigorous. All data are demonstrated in Table 4.

**Table 4:** Association between the exercise intensity among participants during Ramadan and the rest of the year (ROY). The data are represented as numbers (n) and percentages. The association is presented as p-values.

Ramadan	ROY			P-value
Parameters	Low-intensity	Moderate-vigorous intensity	A mixture of different intensities	0.009
Low-intensity	33 (89.2%)	10 (34.5%)	4 (17.4%)	
Moderate-vigorous intensity	4 (10.8%)	19 (65.5%)	5 (21.7%)	
A mixture of different intensities	0 (0.0%)	0 (0.0%)	14 (60.9%)	

Table 5 presents the association between the demographic characteristics of participants and the rate of exercise during Ramadan. A high exercise rate during Ramadan among athletic participants was statistically significant (p = 0.001). On the other hand, factors such as age, gender, marital status, workplace, specialty, and suffering from chronic medical conditions had no impact on the exercise rate during Ramadan.

**Table 5:** Factors associated with the rate of exercise in Ramadan. The data are represented as numbers (n) and percentages. The association is presented as p-values. *: Fisher’s exact test was used if the count in one cell or more was less than 5.

Parameters		Exercise rate per week during Ramadan			P-value*
	Categories	<1 hour	1–3 hours	>3 hours	
Gender	Male	10 (31.3%)	12 (37.5%)	10 (31.3%)	0.772
	Female	22 (38.6%)	20 (35.1%)	15 (26.3%)	
Age	40 years or less	20 (37%)	21 (38.9%)	13 (24.1%)	0.559
	More than 40 years	12 (34.3%)	11 (31.4%)	12 (34.3%)	
Marital status	Married	26 (37.7%)	21 (30.4%)	22 (31.9%)	0.109
	Non-married	6 (30%)	11 (55%)	3 (15%)	
Workplace	Governmental hospitals	25 (40.3%)	21 (33.9%)	16 (25.8%)	0.385
	Private hospitals	3 (23.1%)	7 (53.8%)	3 (23.1%)	
	Both	4 (30.8%)	3 (23.1%)	6 (46.2%)	
Specialty	Physician	15 (41.7%)	10 (27.8%)	11 (30.6%)	0.883
	Surgeon	3 (25%)	5 (41.7%)	4 (33.3%)	
	Nurse	4 (50%)	3 (37.5%)	1 (12.5%)	
	Physical therapist	3 (37.5%)	3 (37.5%)	2 (25%)	
	Others	7 (28%)	11 (44%)	7 (28%)	
Chronic medical illness	Yes	3 (16.7%)	9 (50%)	6 (33.3%)	0.150
	No	29 (40.8%)	23 (32.4%)	19 (26.8%)	
Usual exercise activity	Sedentary	11 (68.8%)	5 (31.3%)	0 (0%)	0.001
	Moderate, very active	21 (30%)	27 (38.6%)	22 (31.4%)	
	Athletic	0 (0%)	0 (0%)	3 (100%)	

This study illustrated that the male gender was significantly associated with moderate or vigorous-intensity exercise during Ramadan. At the same time, the female gender had a significant association with a different mixture of exercise intensity (p = 0.039). The study also showed that moderate or vigorous activity in participants was significantly associated with moderate or vigorous exercise intensity. Meanwhile, different mixtures of exercise intensity during Ramadan were significantly found among athletics (p = 0.002). Data are demonstrated in Table 6.

**Table 6:** Factors associated with the intensity of exercise in Ramadan. The data are represented as numbers (n) and percentages. The association is presented as p-values. *: Fisher’s exact test was used if the count in one cell or more was less than 5.

Parameters		Exercise intensity during Ramadan			P-value*
	Categories	Low-intensity	Moderate-vigorous intensity	A mixture of different intensities	
Gender	Male	18 (56.3%)	13 (40.6%)	1 (3.1%)	0.039
	Female	29 (50.9%)	15 (26.3%)	13 (22.8%)	
Age	40 years or less	27 (50%)	17 (31.5%)	10 (18.5%)	0.643
	More than 40 years	20 (57.1%)	11 (31.4%)	4 (11.4%)	
Marital status	Married	38 (55.1%)	23 (33.3%)	8 (11.6%)	0.137
	Non-married	9 (45%)	5 (25%)	6 (30%)	
Workplace	Governmental hospitals	34 (54.8%)	18 (29%)	10 (16.1%)	0.982
	Private hospitals	6 (46.2%)	5 (38.5%)	2 (15.4%)	
	Both	7 (53.8%)	4 (30.8%)	2 (15.4%)	
Chronic medical illness	Yes	12 (66.7%)	4 (22.2%)	2 (11.1%)	0.419
	No	35 (49.3%)	24 (33.8%)	12 (16.9%)	
Specialty	Physician	20 (55.6%)	13 (36.1%)	3 (8.3%)	0.060
	Surgeon	8 (66.7%)	4 (33.3%)	0 (0%)	
	Nurse	5 (62.5%)	3 (37.5%)	0 (0%)	
	Physical therapist	4 (50%)	3 (37.5%)	1 (12.5%)	
	Others	10 (40%)	5 (20%)	10 (40%)	
Usual exercise activity	Sedentary	14 (87.5%)	1 (6.3%)	1 (6.3%)	0.002
	Moderate, very active	33 (47.1%)	26 (37.1%)	11 (15.7%)	
	Athletic	0 (0%)	1 (33.3%)	2 (66.7%)	

## Discussion

This study demonstrated the exercise habits among HCPs during Ramadan and the rest of the year. The study showed that the intensity of exercise during Ramadan was significantly reduced compared with the intensity level for the rest of the year. On the other hand, the duration of physical activity per year did not significantly change during Ramadan.

Our results are in accordance with prior studies. A study conducted among Malay Muslims and Muslimah at the University Putra Malaysia demonstrated a reduced level of physical activity during Ramadan without mentioning the rate or intensity of exercise [13]. Another study conducted among adolescent soccer players demonstrated a reduction in physical performance during Ramadan [14].

This study showed that time restriction was the most common exercise barrier among HCPs during Ramadan. Similarly, the Malaysian study showed that more time spent on religious and spiritual activities such as prayer might reduce the exercise level during Ramadan [13].

Furthermore, in this study, the usual HCPs’ exercise lifestyle before Ramadan, as sedentary, moderate, severely active, or athletic, had an impact on the rate and intensity of exercise during Ramadan. To our knowledge, the impact of the physical description on exercise habits during Ramadan had not been reported.

This study demonstrated that the male gender was significantly associated with performing moderate-to-severe-intensity exercise during Ramadan. At the same time, another study illustrated the significant reduction of physical exercise among men during Ramadan [15].

This study demonstrated that about half of HCPs had no change in weight during Ramadan. Similarly, a study conducted among adolescent soccer players showed no weight change during Ramadan [14]. In contrast, a study conducted among Tehran University medical students showed a reduction in weight during Ramadan [16]. Meanwhile, Siddiqui et al. illustrated weight increase among healthy men during Ramadan [17].

This study had some limitations. For instance, the study included a small number of participants who were not representative of HCPs in Jeddah, Saudi Arabia. Additionally, the study did not mention several factors that may influence exercise habits, such as changes in work hours, sleep patterns, and dietary intake during Ramadan.

## Conclusions

This study illustrated the reduction in exercise intensity among HCPs during Ramadan. Further studies are recommended to identify the changes in exercise habits during Ramadan and recognize the associated factors and reasons for those changes.
